# Microscopic Analysis and Quality Assessment of Induced Sputum From Children With Pneumonia in the PERCH Study

**DOI:** 10.1093/cid/cix083

**Published:** 2017-05-29

**Authors:** David R. Murdoch, Susan C. Morpeth, Laura L. Hammitt, Amanda J. Driscoll, Nora L. Watson, Henry C. Baggett, W. Abdullah Brooks, Maria Deloria Knoll, Daniel R. Feikin, Karen L. Kotloff, Orin S. Levine, Shabir A. Madhi, Katherine L. O’Brien, J. Anthony G. Scott, Donald M. Thea, Dilruba Ahmed, Juliet O. Awori, Andrea N. DeLuca, Bernard E. Ebruke, Melissa M. Higdon, Possawat Jorakate, Ruth A. Karron, Sidi Kazungu, Geoffrey Kwenda, Lokman Hossain, Sirirat Makprasert, David P. Moore, Azwifarwi Mudau, John Mwaba, Sandra Panchalingam, Daniel E. Park, Christine Prosperi, Rasheed Salaudeen, Aliou Toure, Scott L. Zeger, Stephen R. C. Howie, for the PERCH Study Group, Katherine L. O’Brien, Orin S. Levine, Maria Deloria Knoll, Daniel R. Feikin, Andrea N. DeLuca, Amanda J. Driscoll, Nicholas Fancourt, Wei Fu, Laura L. Hammitt, Melissa M. Higdon, E. Wangeci Kagucia, Ruth A. Karron, Mengying Li, Daniel E. Park, Christine Prosperi, Zhenke Wu, Scott L. Zeger, Nora L. Watson, Jane Crawley, David R. Murdoch, W. Abdullah Brooks, Hubert P. Endtz, Khalequ Zaman, Doli Goswami, Lokman Hossain, Yasmin Jahan, Hasan Ashraf, Stephen R. C. Howie, Bernard E. Ebruke, Martin Antonio, Jessica McLellan, Eunice Machuka, Arifin Shamsul, Syed M. A. Zaman, Grant Mackenzie, J. Anthony G. Scott, Juliet O. Awori, Susan C. Morpeth, Alice Kamau, Sidi Kazungu, Micah Silab Ominde, Karen L. Kotloff, Milagritos D. Tapia, Samba O. Sow, Mamadou Sylla, Boubou Tamboura, Uma Onwuchekwa, Nana Kourouma, Aliou Toure, Shabir A. Madhi, David P. Moore, Peter V. Adrian, Vicky L. Baillie, Locadiah Kuwanda, Azwifarwi Mudau, Michelle J. Groome, Nasreen Mahomed, Henry C. Baggett, Somsak Thamthitiwat, Susan A. Maloney, Charatdao Bunthi, Julia Rhodes, Pongpun Sawatwong, Pasakorn Akarasewi, Donald M. Thea, Lawrence Mwananyanda, James Chipeta, Phil Seidenberg, James Mwansa, Somwe wa Somwe, Geoffrey Kwenda, Trevor P. Anderson, Joanne Mitchell

**Affiliations:** 1 Department of Pathology, University of Otago, and; 2 Microbiology Unit, Canterbury Health Laboratories, Christchurch, New Zealand;; 3 Kenya Medical Research Institute–Wellcome Trust Research Programme, Kilifi;; 4 Department of Infectious Disease Epidemiology, London School of Hygiene & Tropical Medicine, United Kingdom;; 5 Microbiology Laboratory, Middlemore Hospital, Counties Manukau District Health Board, Auckland, New Zealand;; 6 Department of International Health, International Vaccine Access Center, Johns Hopkins Bloomberg School of Public Health, Baltimore and; 7 Emmes Corporation, Rockville, Maryland;; 8 Global Disease Detection Center, Thailand Ministry of Public Health–US Centers for Disease Control and Prevention Collaboration, Nonthaburi;; 9 Division of Global Health Protection, Center for Global Health, Centers for Disease Control and Prevention, Atlanta, Georgia;; 10 Department of International Health, Johns Hopkins Bloomberg School of Public Health, Baltimore, Maryland;; 11 International Centre for Diarrhoeal Disease Research, Bangladesh (icddr,b), Dhaka and Matlab;; 12 Division of Viral Diseases, National Center for Immunization and Respiratory Diseases, Centers for Disease Control and Prevention, Atlanta, Georgia; 13 Division of Infectious Disease and Tropical Pediatrics, Department of Pediatrics, Center for Vaccine Development, Institute of Global Health, University of Maryland School of Medicine, Baltimore;; 14 Bill & Melinda Gates Foundation, Seattle, Washington;; 15 Medical Research Council, Respiratory and Meningeal Pathogens Research Unit and; 16 Department of Science and Technology/National Research Foundation, Vaccine Preventable Diseases Unit, University of the Witwatersrand, Johannesburg, South Africa;; 17 Center for Global Health and Development, Boston University School of Public Health, Massachusetts;; 18 Department of Epidemiology, Johns Hopkins Bloomberg School of Public Health, Baltimore, Maryland;; 19 Medical Research Council Unit, Basse, The Gambia;; 20 Department of International Health, Center for Immunization Research, Johns Hopkins Bloomberg School of Public Health, Baltimore, Maryland;; 21 Department of Biomedical Sciences, School of Health Sciences, University of Zambia, and; 22 Zambia Center for Applied Health Research and Development, Lusaka;; 23 Department of Paediatrics and Child Health, Chris Hani Baragwanath Academic Hospital and University of the Witwatersrand, Johannesburg, South Africa;; 24 Department of Pathology and Microbiology, University Teaching Hospital, Lusaka, Zambia;; 25 Department of Medicine, Center for Vaccine Development, Institute of Global Health, University of Maryland School of Medicine, Baltimore;; 26 Milken Institute School of Public Health, Department of Epidemiology and Biostatistics, George Washington University, District of Columbia;; 27 Medical Microbiology Department, Lagos University Teaching Hospital, Nigeria;; 28 Centre pour le Développement des Vaccins (CVD-Mali), Bamako;; 29 Department of Biostatistics, Johns Hopkins Bloomberg School of Public Health, Baltimore, Maryland;; 30 Department of Paediatrics, University of Auckland, and; 31 Centre for International Health, University of Otago, Dunedin, New Zealand; 32 Johns Hopkins Bloomberg School of Public Health, Baltimore, Maryland; 33 Emmes Corporation, Rockville, Maryland; 34 Nuffield Department of Clinical Medicine, University of Oxford, United Kingdom; 35 University of Otago, Christchurch, New Zealand; 36 icddr,b, Dhaka and Matlab, Bangladesh:; 37 Medical Research Council, Basse, The Gambia; 38 Kenya Medical Research Institute—Wellcome Trust Research Programme, Kilifi; 39 Division of Infectious Disease and Tropical Pediatrics, Department of Pediatrics, Center for Vaccine Development, Institute of Global Health, University of Maryland School of Medicine, Baltimore, and Centre pour le Développement des Vaccins (CVD-Mali), Bamako, Mali; 40 Respiratory and Meningeal Pathogens Research Unit, University of the Witwatersrand, Johannesburg, South Africa; 41 Thailand Ministry of Public Health—US CDC Collaboration, Nonthaburi; 42 Boston University School of Public Health, Boston, Massachusetts and University Teaching Hospital, Lusaka, Zambia; 43 Canterbury Health Laboratory, Christchurch, New Zealand

**Keywords:** pneumonia, induced sputum, quality, children.

## Abstract

**Background.:**

It is standard practice for laboratories to assess the cellular quality of expectorated sputum specimens to check that they originated from the lower respiratory tract. The presence of low numbers of squamous epithelial cells (SECs) and high numbers of polymorphonuclear (PMN) cells are regarded as indicative of a lower respiratory tract specimen. However, these quality ratings have never been evaluated for induced sputum specimens from children with suspected pneumonia.

**Methods.:**

We evaluated induced sputum Gram stain smears and cultures from hospitalized children aged 1–59 months enrolled in a large study of community-acquired pneumonia. We hypothesized that a specimen representative of the lower respiratory tract will contain smaller quantities of oropharyngeal flora and be more likely to have a predominance of potential pathogens compared to a specimen containing mainly saliva. The prevalence of potential pathogens cultured from induced sputum specimens and quantity of oropharyngeal flora were compared for different quantities of SECs and PMNs.

**Results.:**

Of 3772 induced sputum specimens, 2608 (69%) had <10 SECs per low-power field (LPF) and 2350 (62%) had >25 PMNs per LPF, measures traditionally associated with specimens from the lower respiratory tract in adults. Using isolation of low quantities of oropharyngeal flora and higher prevalence of potential pathogens as markers of higher quality, <10 SECs per LPF (but not >25 PMNs per LPF) was the microscopic variable most associated with high quality of induced sputum.

**Conclusions.:**

Quantity of SECs may be a useful quality measure of induced sputum from young children with pneumonia.

Sputum is the lower respiratory specimen most commonly collected from adults as part of the diagnostic workup for community-acquired pneumonia. However, sputum collection is more problematic in children, who typically have difficulty with expectoration [1, 2]. Collection of induced sputum through methods such as hypertonic saline nebulization can potentially overcome this problem.

Specimen quality has a large impact on the interpretation of sputum culture results [3]. Specimens originating from the lower respiratory tract can be contaminated by upper respiratory secretions during the collection process, and some poorly collected specimens may be entirely composed of upper respiratory secretions. Either situation can lead to the incorrect conclusion that an organism colonizing the upper airways is causing pneumonia. Consequently, it has become standard practice for diagnostic laboratories to assess the quality of an expectorated sputum specimen using indicators that suggest it has been obtained from the lower respiratory tract. This involves assessing the number of squamous epithelial cells (SECs) and polymorphonuclear cells (PMNs) in a Gram-stained smear of the specimen [4, 5]. The presence of low numbers of SECs and high numbers of PMNs per low-power field (LPF) have been traditionally regarded as being indicative of a high-quality specimen [6]. Likewise, sputum specimens with relatively low numbers of PMNs and high numbers of SECs are likely to represent oropharyngeal contamination and are typically rejected for routine culture. These quality systems have been developed for expectorated sputum from adults, but have never been formally evaluated for induced sputum samples from children with suspected pneumonia.

This is the first of 5 companion papers in this supplement on induced sputum analysis from the Pneumonia Etiology Research for Child Health (PERCH) study. This article is focused on the assessment of whether pediatric induced sputum specimens are representative of the lower respiratory tract and does not evaluate the utility of induced sputum for diagnostic testing. A specific objective was to identify a quality measure indicating a lower-respiratory tract source that could be applied to induced sputum specimens from children with pneumonia. Other articles in the supplement focus on the usefulness of induced sputum culture, the added value of testing induced sputum by polymerase chain reaction (PCR), safety of induced sputum collection, and utility of induced sputum for diagnosing tuberculosis [7–9].

## METHODS

### Participants

Participants were children aged 1–59 months who were hospitalized with World Health Organization (WHO)–defined severe or very severe pneumonia as part of the PERCH study, a case-control study involving 9 sites in 7 countries from sub-Saharan Africa and South Asia. Details of this study have been described elsewhere [10, 11]. As part of a comprehensive evaluation, induced sputum was collected from cases, ideally before antibiotics were administered.

### Specimen Collection

Induced sputum was obtained at enrollment by study staff following an established methodology [12, 13]. A β-2 agonist was given by a metered dose inhaler 5 minutes prior to nebulization with sterile hypertonic saline (3%–5% sodium chloride) to minimize the risk of bronchospasm. Saline nebulization occurred for at least 10 minutes using a jet nebulizer with a facemask and mixed oxygen flow at a rate of 5–8 L/minute. Percussion of the chest wall was done in children <24 months of age during nebulization, and in older children in the absence of cough. Each quadrant of the posterior aspect of the chest was tapped gently 5–10 times to mobilize lower respiratory secretions and induce a cough in the child. A sterile mucus extracting catheter attached to a suction device was then inserted through the nose into the posterior nasopharynx and sputum was collected into a sterile trap. Suction was applied only once the catheter was in place and not applied during removal of the catheter to avoid aspirating anterior nasal contents. The catheter was flushed with 5 mL sterile normal saline at the end of the procedure, and the specimen was immediately sent to the laboratory for processing.

### Laboratory Methods

Gram-stained smears were made from the most visually purulent portion of each induced sputum specimen. The quality of sputum was assessed by determining the numbers of SECs and PMNs within the following categories: <10, 10–25, or >25 cells per representative (×100) LPF. Microorganisms seen in the smear under high power (×1000) were described according to classic Gram stain morphotypes.

The most purulent portion of each specimen was inoculated onto sheep or horse blood, chocolate, and MacConkey agars, streaked out using a standard 4-quadrant streaking method, and incubated at 35°C for 48 hours. Cultures were examined at 24 hours and 48 hours, and predominant organisms were identified and quantified according to the furthest quadrant with visible colonies (first quadrant, scanty; second quadrant, 1+; third quadrant, 2+; fourth quadrant, 3+). Background mixed oropharyngeal flora (including viridans streptococci, commensal *Neisseria*, coagulase-negative staphylococci, yeasts [except *Cryptococcus*], diphtheroids, and *Capnocytophaga*) were quantified as a group but not identified further.

Medical laboratory scientists processed the specimens at each site, and efforts were made to standardize these methods across all study sites through uniform standard operating procedures, on-site training, and internal and external quality checks [14] (including participation in the Royal College of Pathologists of Australasia’s Quality Assurance Programme).

### Study Definitions

Sputum culture results were interpreted using the following 6 increasingly more rigorous interpretive criteria for each organism identified:

1. Organism isolated in any quantity;
2. Organism isolated in any quantity and with compatible Gram stain morphotype;
3. Organism isolated as the predominant organism;
4. Organism isolated as the predominant organism and with compatible Gram stain morphotype;
5. Organism isolated in quantities of 2+ or 3+;
6. Organism isolated in quantities of 2+ or 3+ and with compatible Gram stain morphotype.

Prior antimicrobial therapy was defined as (1) antibiotic activity in serum by bioassay, or (2) documented administration of antibiotics before induced sputum sample collection [15].

Chest radiographs from each child were read by a panel of radiologists and pediatricians trained in the standardized interpretation of pediatric chest radiographs [16]. Chest radiographs were classified as either consolidation, other infiltrate, both consolidation and other infiltrate, normal, or uninterpretable.

### Statistical Analysis

As there are no suitable gold standards to assess sputum quality, we identified variables that were likely markers of sputum quality. We hypothesized that a specimen representative of the lower respiratory tract will contain smaller quantities of oropharyngeal flora and larger quantities of potential pathogens compared to a poor-quality specimen containing mainly saliva.

The prevalence of potential pathogens cultured from induced sputum specimens was compared across the 6 interpretive criteria and for different quantities of SECs and PMNs. The quantity of oropharyngeal flora was also compared for different quantities of SECs and PMNs.

To characterize potential correlates of poorer-quality specimens, we used logistic regression models of clinical characteristics (prior antimicrobial use, radiographic pneumonia, and human immunodeficiency virus infection), SEC quantity, and PMN quantity as predictors of higher oropharyngeal flora quantities. Associations between each clinical characteristic and oropharyngeal flora quantity were estimated by odds ratios (unadjusted and adjusted for all evaluated characteristics and PERCH site). Oropharyngeal flora quantity was evaluated as quantity greater or equal to 2+ or 3+ vs lower quantity or not present.

### Ethical Considerations

The study protocol was approved by the institutional review board or ethics committee at each of the 7 institutions and at the Johns Hopkins School of Public Health. Parents or guardians of participants provided written informed consent.

## RESULTS

Induced sputum culture results were available for analysis from 3772 of 4232 (89.1%) children enrolled in PERCH; 2695 (71.4%) had severe pneumonia and 1077 (28.6%) very severe pneumonia: 518 from Bangladesh, 596 from The Gambia, 592 from Kenya, 544 from Mali, 824 from South Africa, 191 from Thailand, and 507 from Zambia. The median age of the children was 8 months (interquartile range, 3–16 months), and 1579 (41.9%) were female; 2833 (75.1%) had evidence of receipt of antimicrobials before collection of induced sputum.

Table 1 shows the characteristics of the induced sputum specimens by demographic and clinical variables. There was variability in the quality of specimens across study sites, with large numbers of SECs reported in a higher proportion of cases from South Africa. Detection of 4 major potential pathogens (*Streptococcus pneumoniae*, *Haemophilus influenzae*, *Moraxella catarrhalis*, *Staphylococcus aureus*) was greater in specimens from children without evidence of prior antibiotic use. Otherwise, there was little variation in sputum characteristics for most variables.

**Table 1.** Characterization of Induced Sputum Specimens From Children Aged 1–59 Months With World Health Organization–Defined Severe or Very Severe Pneumonia by Case Clinical Factors (N = 3772)

		PMNs per LPF			SECs per LPF			Oropharyngeal Flora^a^				Any Potential Pathogen by Sputum Interpretive Criteria^b^					
		<10	10–25	>25	<10	10–25	>25	Scanty	1+	2+	3+	1	2	3	4	5	6
All		736 (19.5)	686 (18.2)	2350 (62.3)	2608 (69.1)	740 (19.6)	424 (11.2)	684 (18.1)	891 (23.6)	789 (20.9)	661 (17.5)	2376 (63)	1299 (34.4)	2307 (61.2)	1268 (33.6)	1450 (38.4)	1008 (26.7)
Age	<6 mo	345 (22.5)	293 (19.1)	893 (58.3)	995 (65)	344 (22.5)	192 (12.5)	276 (18)	396 (25.9)	324 (21.2)	236 (15.4)	924 (60.4)	487 (31.8)	883 (57.7)	468 (30.6)	554 (36.2)	368 (24)
	6–11 mo	143 (16.7)	161 (18.8)	552 (64.5)	586 (68.5)	158 (18.5)	112 (13.1)	145 (16.9)	188 (22)	188 (22)	161 (18.8)	536 (62.6)	310 (36.2)	519 (60.6)	303 (35.4)	338 (39.5)	243 (28.4)
	12–23 mo	146 (17.1)	137 (16)	572 (66.9)	623 (72.9)	156 (18.2)	76 (8.9)	160 (18.7)	186 (21.8)	178 (20.8)	164 (19.2)	557 (65.1)	299 (35)	552 (64.6)	296 (34.6)	335 (39.2)	237 (27.7)
	>23 mo	102 (19.2)	95 (17.9)	333 (62.8)	404 (76.2)	82 (15.5)	44 (8.3)	103 (19.4)	121 (22.8)	99 (18.7)	100 (18.9)	359 (67.7)	203 (38.3)	353 (66.6)	201 (37.9)	223 (42.1)	160 (30.2)
HIV	Negative	597 (18.4)	590 (18.2)	2063 (63.5)	2239 (68.9)	649 (20)	362 (11.1)	565 (17.4)	783 (24.1)	687 (21.1)	581 (17.9)	2031 (62.5)	1128 (34.7)	1983 (61)	1105 (34)	1249 (38.4)	877 (27)
	Positive	22 (10.8)	30 (14.8)	151 (74.4)	125 (61.6)	37 (18.2)	41 (20.2)	23 (11.3)	33 (16.3)	52 (25.6)	36 (17.7)	115 (56.7)	53 (26.1)	107 (52.7)	50 (24.6)	63 (31)	41 (20.2)
Sex	Male	433 (19.7)	402 (18.3)	1358 (61.9)	1532 (69.9)	433 (19.7)	228 (10.4)	416 (19)	518 (23.6)	456 (20.8)	397 (18.1)	1423 (64.9)	793 (36.2)	1379 (62.9)	774 (35.3)	876 (39.9)	613 (28)
	Female	303 (19.2)	284 (18)	992 (62.8)	1076 (68.1)	307 (19.4)	196 (12.4)	268 (17)	373 (23.6)	333 (21.1)	264 (16.7)	953 (60.4)	506 (32)	928 (58.8)	494 (31.3)	574 (36.4)	395 (25)
Severity	Severe	521 (19.3)	450 (16.7)	1724 (64)	1869 (69.4)	521 (19.3)	305 (11.3)	464 (17.2)	614 (22.8)	590 (21.9)	515 (19.1)	1794 (66.6)	1056 (39.2)	1754 (65.1)	1032 (38.3)	1151 (42.7)	837 (31.1)
	Very severe	215 (20)	236 (21.9)	626 (58.1)	739 (68.6)	219 (20.3)	119 (11)	220 (20.4)	277 (25.7)	199 (18.5)	146 (13.6)	582 (54)	243 (22.6)	553 (51.3)	236 (21.9)	299 (27.8)	171 (15.9)
Site	Kenya	81 (13.7)	191 (32.3)	320 (54.1)	514 (86.8)	74 (12.5)	4 (0.7)	161 (27.2)	127 (21.5)	60 (10.1)	116 (19.6)	380 (64.2)	71 (12)	376 (63.5)	71 (12)	219 (37)	68 (11.5)
	The Gambia	272 (45.6)	94 (15.8)	230 (38.6)	439 (73.7)	121 (20.3)	36 (6)	125 (21)	106 (17.8)	149 (25)	108 (18.1)	534 (89.6)	438 (73.5)	533 (89.4)	438 (73.5)	440 (73.8)	382 (64.1)
	Mali	180 (33.1)	93 (17.1)	271 (49.8)	339 (62.3)	141 (25.9)	64 (11.8)	154 (28.3)	165 (30.3)	107 (19.7)	33 (6.1)	327 (60.1)	184 (33.8)	293 (53.9)	167 (30.7)	148 (27.2)	107 (19.7)
	Zambia	47 (9.3)	95 (18.7)	365 (72)	416 (82.1)	60 (11.8)	31 (6.1)	115 (22.7)	100 (19.7)	50 (9.9)	12 (2.4)	320 (63.1)	71 (14)	314 (61.9)	69 (13.6)	105 (20.7)	37 (7.3)
	South Africa	82 (10)	98 (11.9)	644 (78.2)	373 (45.3)	202 (24.5)	249 (30.2)	0 (0)	196 (23.8)	260 (31.6)	220 (26.7)	374 (45.4)	220 (26.7)	353 (42.8)	209 (25.4)	258 (31.3)	165 (20)
	Thailand	23 (12)	36 (18.8)	132 (69.1)	178 (93.2)	11 (5.8)	2 (1)	57 (29.8)	83 (43.5)	23 (12)	5 (2.6)	123 (64.4)	64 (33.5)	122 (63.9)	63 (33)	49 (25.7)	40 (20.9)
	Bangladesh	51 (9.8)	79 (15.3)	388 (74.9)	349 (67.4)	131 (25.3)	38 (7.3)	72 (13.9)	114 (22)	140 (27)	167 (32.2)	318 (61.4)	251 (48.5)	316 (61)	251 (48.5)	231 (44.6)	209 (40.3)
Prior antibiotics	No	246 (29.2)	137 (16.3)	459 (54.5)	598 (71)	165 (19.6)	79 (9.4)	118 (14)	169 (20.1)	216 (25.7)	195 (23.2)	741 (88)	588 (69.8)	731 (86.8)	585 (69.5)	598 (71)	503 (59.7)
	Yes	466 (16.4)	529 (18.7)	1838 (64.9)	1937 (68.4)	560 (19.8)	336 (11.9)	552 (19.5)	701 (24.7)	550 (19.4)	437 (15.4)	1554 (54.9)	650 (22.9)	1496 (52.8)	622 (22)	777 (27.4)	447 (15.8)
CXR positive^c^	No	321 (20.9)	282 (18.3)	936 (60.8)	1111 (72.2)	279 (18.1)	149 (9.7)	283 (18.4)	368 (23.9)	310 (20.1)	283 (18.4)	1020 (66.3)	592 (38.5)	997 (64.8)	580 (37.7)	634 (41.2)	455 (29.6)
	Yes	330 (19)	318 (18.3)	1091 (62.7)	1162 (66.8)	361 (20.8)	216 (12.4)	306 (17.6)	415 (23.9)	380 (21.9)	304 (17.5)	1053 (60.6)	554 (31.9)	1020 (58.7)	539 (31)	644 (37)	438 (25.2)

Denominator for the All row is N = 3772; denominator for subsequent rows corresponds to the number of cases in that subgroup.

Abbreviations: CXR, chest radiograph; HIV, human immunodeficiency virus; LPF, low-power field; PMNs, polymorphonuclear cells; SECs, squamous epithelial cells.

^**a**^Quantity of oropharyngeal flora was recorded in 3677 (97%) sputum samples; no oropharyngeal flora was reported in 652 (17.7%) sputum samples.

^**b**^Number of children with any potential pathogen (defined as *Streptococcus pneumoniae*, *Haemophilus influenzae*, *Staphylococcus aureus*, or *Moraxella catarrhalis*) by sputum interpretive criteria: 1, organism present in any amount; 2, present in any amount with compatible Gram stain morphotype; 3, present as the predominant organism; 4, present as the predominant organism with compatible Gram stain morphotype; 5, present in quantities ≥2+; 6, present in quantities ≥2+ with compatible Gram stain morphotype. Sputum interpretive criteria are not mutually exclusive and children will appear in multiple columns.

^c^Chest radiograph positive defined as radiographic evidence of pneumonia (consolidation and/or other infiltrates).

Over two-thirds of samples had <10 SECs per LPF and a similar proportion had >25 PMNs per LPF (Table 2), quantities traditionally associated with high-quality sputum samples among adult populations. A similar pattern was observed when the analysis was restricted to cases with chest radiographic changes (Supplementary Table 1*A*).

**Table 2.** Comparison of PMN and SEC Quantity in Induced Sputum Samples From Children Aged 1–59 Months With World Health Organization–Defined Severe or Very Severe Pneumonia

No. of SECs per LPF	No. of PMNs per LPF						All	
	>25		10–25		<10			
<10	1553	(41.2)	502	(13.3)	553	(14.7)	2608	(69.1)
10–25	478	(12.7)	137	(3.6)	125	(3.3)	740	(19.6)
>25	319	(8.5)	47	(1.2)	58	(1.5)	424	(11.2)
All	2350	(62.3)	686	(18.2)	736	(19.5)	3772	(100.0)

Data are presented as No. (%). Percentages represent percentage of total specimens among cases in whom induced sputum was collected and had available culture results (N = 3772).

Abbreviations: LPF, low-power field; PMNs, polymorphonuclear cells; SECs, squamous epithelial cells.

Table 3 shows the distribution of organisms cultured from sputum samples using the 6 different interpretive criteria. *Haemophilus influenzae*, *S. pneumoniae*, and *M. catarrhalis* were the predominant organisms isolated. The prevalence of all organisms declined with progressively more rigorous interpretive criteria, as expected.

**Table 3.** Prevalence of Bacteria by Sputum Culture Interpretive Criteria in Induced Sputum Samples From Children Aged 1–59 Months With World Health Organization–Defined Severe or Very Severe Pneumonia (N = 3772)

	Potential Pathogens, No. (%^b^)								
Sputum Culture Interpretive Criteria^a^	Spn	Saur	Oth Str^c^	Hinf	Mcat	Entrb^d^	Mgnr	Ognr^e^	Paer^e^
Organism present in any amount	1095 (29.0)	387 (10.3)	35 (0.9)	1429 (37.9)	1025 (27.2)	422 (11.2)	165 (4.4)	119 (3.2)	27 (0.7)
Present in any amount with compatible Gram stain morphotype	947 (25.1)	179 (4.7)	28 (0.7)	1029 (27.3)	781 (20.7)	185 (4.9)	120 (3.2)	58 (1.5)	19 (0.5)
Present as the predominant organism	860 (22.8)	291 (7.7)	27 (0.7)	1138 (30.2)	825 (21.9)	329 (8.7)	95 (2.5)	94 (2.5)	24 (0.6)
Present as the predominant organism with compatible Gram stain morphotype	744 (19.7)	141 (3.7)	23 (0.6)	794 (21.0)	629 (16.7)	145 (3.8)	68 (1.8)	47 (1.2)	16 (0.4)
Present in quantities ≥2+	683 (18.1)	182 (4.8)	0 (0.0)	819 (21.7)	639 (16.9)	91 (2.4)	39 (1.0)	21 (0.6)	16 (0.4)
Present in quantities ≥2+ with compatible Gram stain morphotype	626 (16.6)	92 (2.4)	0 (0.0)	613 (16.3)	535 (14.2)	50 (1.3)	23 (0.6)	13 (0.3)	11 (0.3)

Abbreviations: Entrb, Enterobacteriaceae; Hinf, *Haemophilus influenzae*; Mcat*, Moraxella catarrhalis*; Mgnr, mixed gram-negative rods; Ognr, other nonfermentative gram-negative rods; Oth Str, other streptococci and enterococci; Paer, *Pseudomonas aeruginosa*; Saur, *Staphylococcus aureus*; Spn, *Streptococcus pneumoniae*.

^a^Sputum culture interpretive criteria are not mutually exclusive; children may appear in multiple criteria.

^b^All percentages are based on total number of induced sputum specimens (N = 3772).

^c^Other streptococci and enterococci includes streptococci (other than *S. pneumoniae*) and enterococci species.

^d^Enterobacteriaceae includes *Escherichia coli*, *Enterobacter* species, *Klebsiella* species, *Citrobacter* species, and *Serratia* species, excluding mixed gram-negative rods.

^e^Other nonfermentative gram-negative rods includes *Acinetobacter* species and *Pseudomonas* species. *Pseudomonas aeruginosa* also reported separately.

**Figure 1.**
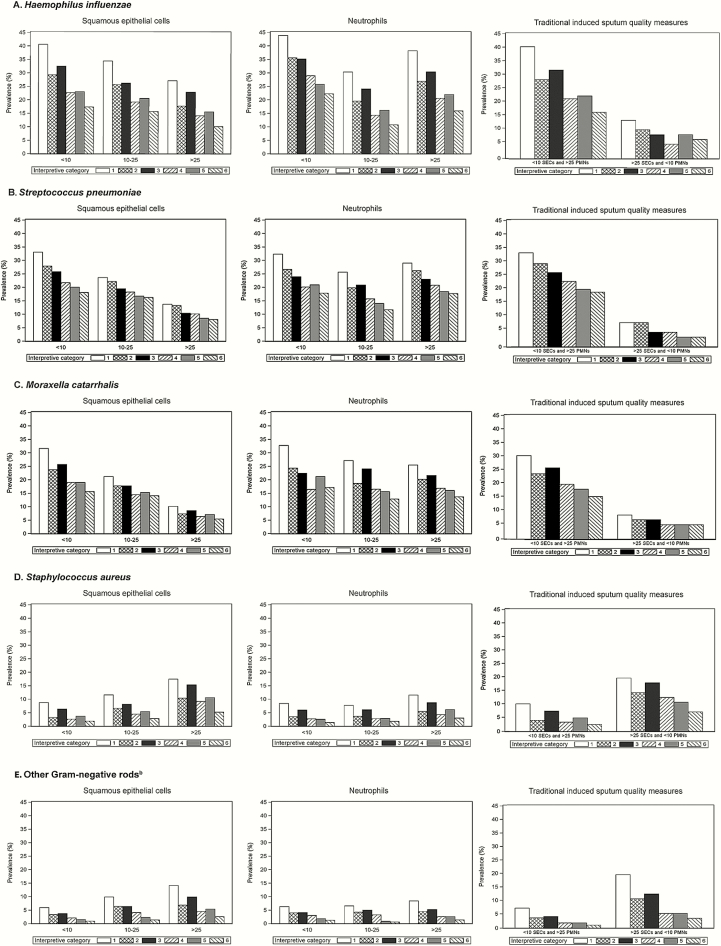
*A–E*, Prevalence of organisms by induced sputum culture interpretive criteria^a^ and induced sputum quality variables in children aged 1–59 months with World Health Organization–defined severe or very severe pneumonia. Sputum interpretive criteria: 1, organism present in any amount; 2, present in any amount with compatible Gram stain morphotype; 3, present as the predominant organism; 4, present as the predominant organism with compatible Gram stain morphotype; 5, present in quantities ≥2+; 6, present in quantities ≥2+ with compatible Gram stain morphotype. ^a^Sputum interpretive criteria are not mutually exclusive and children will appear in multiple columns. ^b^Other nonfermentative gram-negative rods include *Acinetobacter* species and *Pseudomonas* species. Abbreviations: PMNs, polymorphonuclear cells; SECs, squamous epithelial cells.

Figure 1 shows the prevalence of the 5 major organism groupings with differing culture interpretive criteria and with varying quantities of SECs and PMNs. The prevalence of *H. influenzae*, *S. pneumoniae*, and *M. catarrhalis* decreased with increasing numbers of SECs. The same relationship was not observed for other gram-negative bacteria or *S. aureus*, for which there was a slight increase in prevalence with increasing numbers of SECs. The prevalence of all organisms remained relatively unchanged with varying numbers of PMNs. These patterns were similar when the analysis was restricted to cases with chest radiographic changes (Supplementary Figure 1*A*). The findings were also similar when the analysis was stratified by prior antibiotic use, although organism prevalence was lower in cases with prior antibiotic use (Supplementary Figure 1*B* and 1*C*).

**Figure 2.**
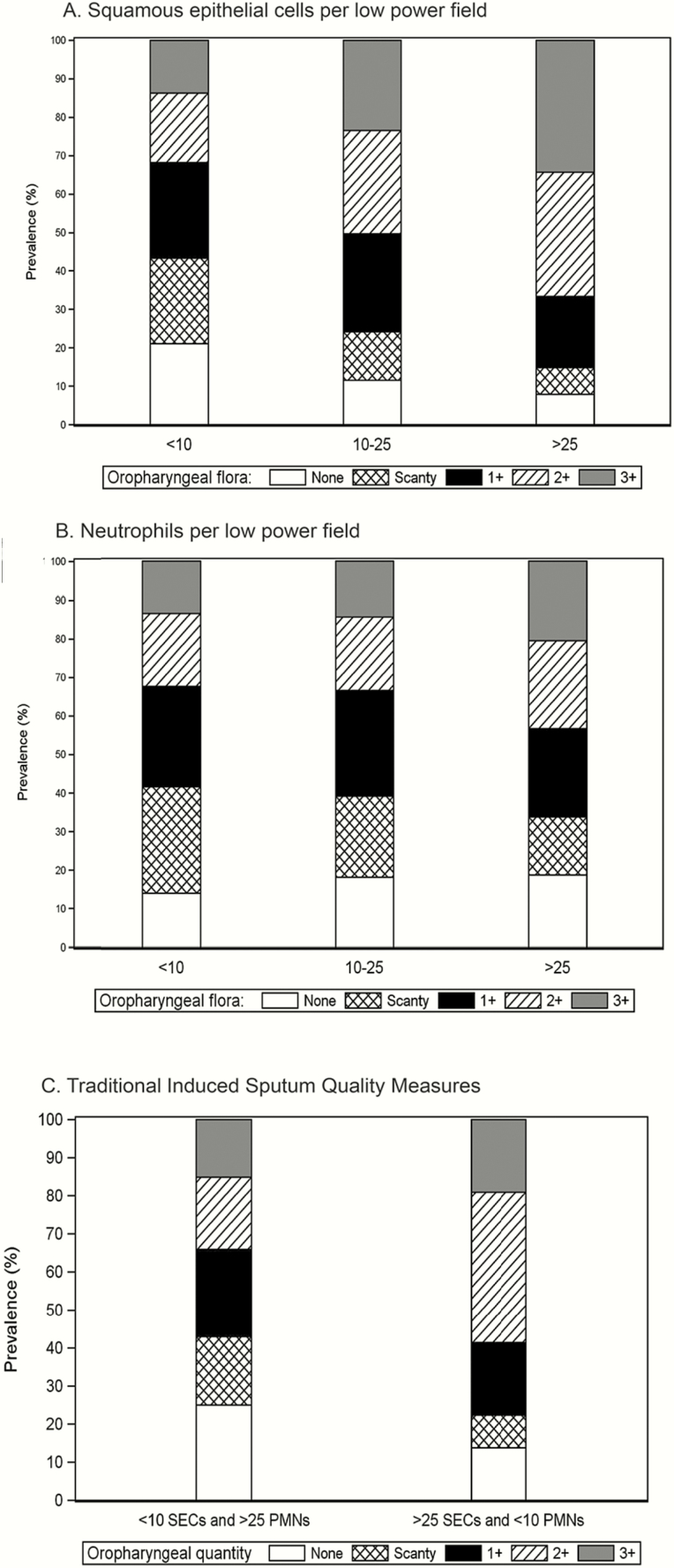
*A–C*, Quantity of oropharyngeal flora in induced sputum in children aged 1–59 months with World Health Organization–defined severe or very severe pneumonia by induced sputum quality variables (N = 3772). Abbreviations: PMNs, polymorphonuclear cells; SECs, squamous epithelial cells.

Quantity of oropharyngeal flora was recorded in 3677 (97%) sputum samples, of which 661 (18%) reported 3+ and 652 (18%) reported no oropharyngeal flora (Table 1). The quantity of oropharyngeal flora increased with the presence of greater numbers of SECs, but there was no clear association with numbers of PMNs (Figure 2). The findings were similar when restricted to radiographic pneumonia cases and when stratified by prior antibiotic use (Supplementary Figure 2*A–C*).

Table 4 shows the analysis of variables associated with high quantities of oropharyngeal flora. Sputum specimens with fewer SECs were associated with a lower odds of culturing larger quantities of oropharyngeal flora. PMNs >25 per LPF was also associated with an increased odds of culturing larger quantities of oropharyngeal flora, but the effect size was smaller and (unlike with SECs) there was not an increasing trend across PMN categories.

**Table 4.** Associations of Clinical and Induced Sputum Characteristics With 2+/3+ Oropharyngeal Flora in Children Aged 1–59 Months With World Health Organization–Defined Severe or Very Severe Pneumonia

	2+/3+ OROF	
Induced Sputum Characteristic	Unadjusted OR (95% CI)	Multivariable Model^a^ AOR (95% CI)
SECs per LPF		
>25 (reference)	1.00	1.00
10–25	0.57 (.40–.65)	0.62 (.46–.84)
<10	0.23 (.19–.29)	0.31 (.23–.41)
PMNs per LPF		
<10 (reference)	1.00	1.00
10–25	1.05 (.84–1.31)	1.04 (.78–1.38)
>25	1.60 (1.34–1.91)	1.52 (1.21–1.92)
Prior antibiotic use^b^	0.58 (.50–.68)	0.72 (.57–.93)
CXR positive^c^	0.94 (.79–1.13)	0.97 (.82–1.15)
HIV positive	1.19 (.89–1.58)	1.51 (1.02–2.23)

Abbreviations: AOR, adjusted odds ratio; CI, confidence interval; CXR, chest radiograph; HIV, human immunodeficiency virus; LPF, low-power field; OR, odds ratio; OROF, oropharyngeal flora; PMNs, polymorphonuclear cells; SECs, squamous epithelial cells.

^a^Adjusted for all other characteristics included in the model and Pneumonia Etiology Research for Child Health (PERCH) site.

^b^Prior antibiotic use defined as serum bioassay positive, antibiotics received at referral hospital, or administered before induced sputum specimen collection.

^c^CXR positive defined as any abnormal CXR result (consolidation and/or other infiltrate).

## DISCUSSION

The key finding from this study is that <10 SECs per LPF was the best measure of induced sputum quality in young children with pneumonia, using high quantity of oropharyngeal flora and low prevalence of potential pathogens as markers of poorer quality. It was also notable that a large proportion (69.1%) of induced sputum samples met this criterion for good quality. If this criterion is an accurate marker of good quality, this finding implies that a large proportion of induced sputum specimens in this study were actually obtained from the lower respiratory tract.

Criteria used by diagnostic laboratories to identify microscopically high-quality sputum specimens from adults were derived from expert opinion, supported by limited data using surrogate markers of quality such as the quantity of oropharyngeal flora [4, 5]. Sputum with <10 SECs and >25 PMNs per LPF have long been regarded as ideal [17], although the requirement for large numbers of PMNs has been questioned given that some pneumonias are not necessarily associated with production of purulent sputum [18]. Indeed, the sentinel study by Murray and Washington indicated that <10 SECs was the key quality measure, and that the presence of leukocytes did not influence the quality interpretation when substantial numbers of SECs were present [5]. Our findings support the application of <10 SECs as a quality measure for induced sputum specimens from children as well. The reason for the association between >25 PMNs and increased amounts of oropharyngeal flora is unclear, although the effect size was small.

A large amount of cellular material was obtained from most induced sputum samples in this study. More than two-thirds of specimens had <10 SECs per LPF and a similar proportion had >25 PMNs per LPF (40% had both <10 SECs and >25 PMNs per LPF). These findings are similar to those from other childhood pneumonia studies that collected induced sputa [19, 20], and are similar to that reported for expectorated sputum from adults with pneumonia [21, 22]. Our initial concern that the use of the saline flush in the induced sputum collection process may dilute the specimen is likely unwarranted, and probably mitigated by the use of the most purulent portion of the specimen for making the Gram stain smear.

The study has several limitations. Most importantly, we lacked a gold standard for good-quality sputum obtained from the lower respiratory tract and, instead, relied on surrogate markers such as quantity of background oropharyngeal flora. While specimens from the oropharynx are more likely to contain large amounts of oropharyngeal flora, true lower respiratory specimens will also contain normal commensals from the upper airways through contamination in the collection process. The exact relationship between quantities of oropharyngeal flora in upper and lower airways is unknown. Second, as expected, we found evidence that antibiotic use before specimen collection affects culture findings. We accounted for the influence of antibiotics in the analyses, although our imperfect definition of prior antibiotic use may have failed to identify cases who had received antibiotics [15]. Third, despite efforts to standardize methods across sites through training, uniform standard operating procedures, and internal and external quality checks, there may still be variations in the reporting of sputum cultures and Gram stain smears between scientists and between sites. There was variability in findings between sites (Table 1), and it is uncertain the degree to which this reflects true differences in the patient populations and whether there is a contribution from interobserver variability.

Despite these limitations, the results of this study indicate that good-quality sputum specimens can be collected from children with pneumonia through saline nebulization induction, and that analysis should be restricted to specimens with <10 SECs per LPF on Gram stain smear. Although our analysis relied on culture results, this restriction identifies characteristics of sputum specimens most likely to be derived from the lower airways and, therefore, the same criterion could also apply to other testing methods such as PCR. Subsequent analyses will further explore the utility of induced sputum in diagnosing pneumonia etiology [9, 23].

## Supplementary Data

Supplementary materials are available at *Clinical Infectious Diseases* online. Consisting of data provided by the author to benefit the reader, the posted materials are not copyedited and are the sole responsibility of the author, so questions or comments should be addressed to the corresponding author.

## Supplementary Material

DAP_1A_Supplementary_Tables_and_Figures_18Nov2016

## Notes

***Author contributions.*** D. R. M. led the analysis, interpreted results, and drafted the manuscript. N. L. W., S. C. M., D. R. F., L. L. H., and S. R. C. H. participated in the analysis and interpretation of results and drafting of the manuscript. O. S. L., K. L. O., D. R. F., D. R. M., M. D. K., L. L. H., H. C. B., W. A. B., S. R. C. H., K. K. L., S. A. M., J. A. G. S., D. M. T., S. C. M., and R. A. K. conceived and designed the study and supervised study conduct. D. A., J. O. A., A. N. D., B. E. E., M. M. H., P. J., S. K., G. K., L. H., S. M., D. P. M., A. M., J. M., S. P., D. E. P., C. P., R. S., and A. T. were involved in study conduct, data collection, and/or data management. S. L. Z. provided expert statistical guidance. All authors reviewed and approved the manuscript. D. R. M. had final responsibility for the decision to submit for publication.

***Acknowledgments.*** We are grateful to the members of the original study teams for running the studies and collecting data. We would also like to acknowledge members of the following group who contributed to the study design, conduct, and analysis phases of PERCH (see Supplementary Materials for full list of names): Pneumonia Methods Working Group, PERCH Expert Group, PERCH Contributors, and the PERCH Chest Radiograph Reading Panel. We offer sincere thanks to the patients and families who participated in this study.

***PERCH Study Group.*** Johns Hopkins Bloomberg School of Public Health, Baltimore, Maryland: Katherine L. O’Brien (principal investigator [PI]), Orin S. Levine (Former PI, current affiliation Bill & Melinda Gates Foundation, Seattle, Washington), Maria Deloria Knoll (co-PI), Daniel R. Feikin (joint affiliation with Centers for Disease Control and Prevention [CDC], Atlanta, Georgia), Andrea N. DeLuca, Amanda J. Driscoll, Nicholas Fancourt, Wei Fu, Laura L. Hammitt, Melissa M. Higdon, E. Wangeci Kagucia, Ruth A. Karron, Mengying Li, Daniel E. Park, Christine Prosperi, Zhenke Wu, Scott L. Zeger; Emmes Corporation, Rockville, Maryland: Nora L. Watson; Nuffield Department of Clinical Medicine, University of Oxford, United Kingdom: Jane Crawley; University of Otago, Christchurch, New Zealand: David R. Murdoch; icddr,b, Dhaka and Matlab, Bangladesh: W. Abdullah Brooks (site PI), Hubert P. Endtz, Khalequ Zaman, Doli Goswami, Lokman Hossain, Yasmin Jahan, Hasan Ashraf; Medical Research Council, Basse, The Gambia: Stephen R. C. Howie (site PI), Bernard E. Ebruke, Martin Antonio, Jessica McLellan, Eunice Machuka, Arifin Shamsul, Syed M.A. Zaman, Grant Mackenzie; Kenya Medical Research Institute–Wellcome Trust Research Programme, Kilifi: J. Anthony G. Scott (site PI and PERCH co-PI), Juliet O. Awori, Susan C. Morpeth, Alice Kamau, Sidi Kazungu, Micah Silab Ominde; Division of Infectious Disease and Tropical Pediatrics, Department of Pediatrics, Center for Vaccine Development, Institute of Global Health, University of Maryland School of Medicine, Baltimore, and Centre pour le Développement des Vaccins (CVD-Mali), Bamako, Mali: Karen L. Kotloff (site PI), Milagritos D. Tapia, Samba O. Sow, Mamadou Sylla, Boubou Tamboura, Uma Onwuchekwa, Nana Kourouma, Aliou Toure; Respiratory and Meningeal Pathogens Research Unit, University of the Witwatersrand, Johannesburg, South Africa: Shabir A. Madhi (site PI), David P. Moore, Peter V. Adrian, Vicky L. Baillie, Locadiah Kuwanda, Azwifarwi Mudau, Michelle J. Groome, Nasreen Mahomed; Thailand Ministry of Public Health–US CDC Collaboration, Nonthaburi: Henry C. Baggett (site PI), Somsak Thamthitiwat, Susan A. Maloney (former site PI), Charatdao Bunthi, Julia Rhodes, Pongpun Sawatwong, Pasakorn Akarasewi (site co-PI, Ministry of Public Health); Boston University School of Public Health, Boston, Massachusetts and University Teaching Hospital, Lusaka, Zambia: Donald M. Thea (site PI), Lawrence Mwananyanda, James Chipeta, Phil Seidenberg, James Mwansa, Somwe wa Somwe, Geoffrey Kwenda. Canterbury Health Laboratory, Christchurch, New Zealand: Trevor P. Anderson, Joanne Mitchell.

***Financial support.*** PERCH was supported by the Bill & Melinda Gates Foundation (grant number 48968 to the International Vaccine Access Center, Department of International Health, Johns Hopkins Bloomberg School of Public Health). J. A. G. S. was supported by a clinical fellowship from The Wellcome Trust of Great Britain (award number 098532).

***Supplement sponsorship.*** This article appears as part of the supplement “Pneumonia Etiology Research for Child Health (PERCH): Foundational Basis for the Primary Etiology Results,” sponsored by a grant from the Bill & Melinda Gates Foundation to the PERCH study of Johns Hopkins Bloomberg School of Public Health, Baltimore, Maryland.

***Potential conﬂicts of interest.*** M. D. K. has received funding for consultancies from Merck, Pfizer, and Novartis, and grant funding from Merck. L. L. H. has received grant funding from Pfizer and GlaxoSmithKline. K. L. K. has received grant funding from Merck Sharp & Dohme. S. A. M. has received honoraria for advisory board membership from the Bill & Melinda Gates Foundation, Pfizer, Medimmune, and Novartis; has received institutional grants from GSK, Novartis, Pfizer, Minervax, and the Bill & Melinda Gates Foundation; and has served on speaker’s bureaus for Sanofi Pasteur and GSK. K. L. O. has received grant funding from GSK and Pfizer and participates on technical advisory boards for Merck, Sanofi Pasteur, PATH, Affinivax, and ClearPath. All other authors report no potential conflicts. All authors have submitted the ICMJE Form for Disclosure of Potential Conflicts of Interest. Conflicts that the editors consider relevant to the content of the manuscript have been disclosed.
