# Supplementary material for: Microscopic Analysis and Quality Assessment of Induced Sputum From Children With Pneumonia in the PERCH Study
Source: Clin Infect Dis. 2017 May 29;64(Suppl 3):S271–9. doi: 10.1093/cid/cix083 (PMC5447851; doi:10.1093/cid/cix083)
Supplement: DAP_1A_Supplementary_Tables_and_Figures_18Nov2016 [file cix083_suppl_DAP_1A_Supplementary_Tables_and_Figures_18Nov2016.pdf]

Supplementary Table 1a. Comparison of neutrophil and epithelial cell quantity in induced sputum samples from children aged 1-59 months with WHO-defined severe or very severe pneumonia, CXR+ Cases

	**Number of neutrophils per low power field**							
	**>25**		**10-25**		**<10**		**All**	
	**N**	**%**	**N**	**%**	**N**	**%**	**N**	**%**
**Number epithelial cells per low power field**								
**<10**	694	39.9	233	13.4	235	13.5	1162	66.8
**10-25**	232	13.3	65	3.7	64	3.7	361	20.8
**>25**	165	9.5	20	1.2	31	1.8	216	12.4
**All**	1091	62.7	318	18.3	330	19	1739	100.0

Percentages represent percent of total specimens among subset of cases (N=1739).
Abbreviations: WHO, World Health Organization.
CXR+ defined as radiographic evidence of pneumonia (consolidation and/or other infiltrates).

Supplementary Figure 1a. Prevalence of organisms by induced sputum culture interpretive criteria and induced sputum quality variables[a] in children aged 1-59 months with WHO-defined severe or very severe pneumonia, CXR+ Cases
(N=1739)

A. *H. influenzae*, Epithelial cells per LPF

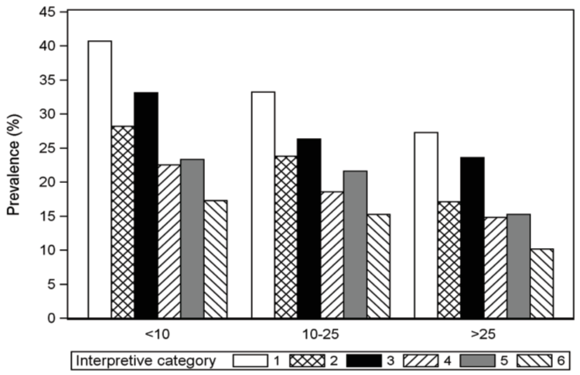

A. *H. influenzae*, Neutrophils per LPF

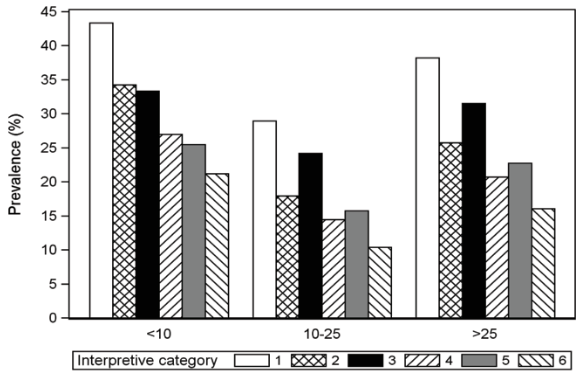

B. *S. pneumoniae*, Epithelial cells per LPF

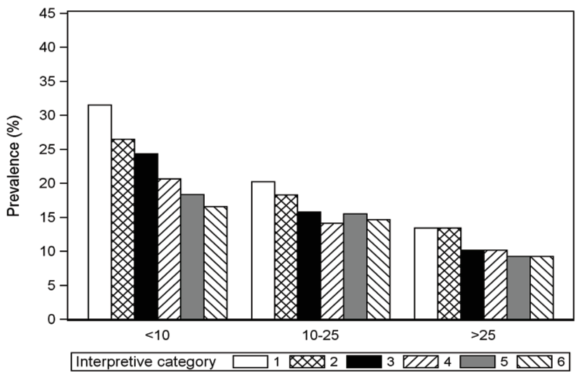

B. *S. pneumoniae,* Neutrophils per LPF

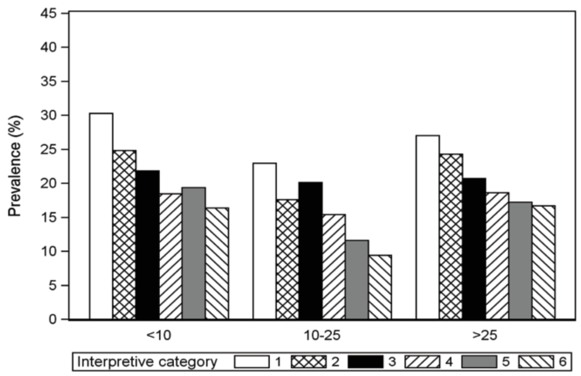

C. *M. catarrhalis*, Epithelial cells per LPF

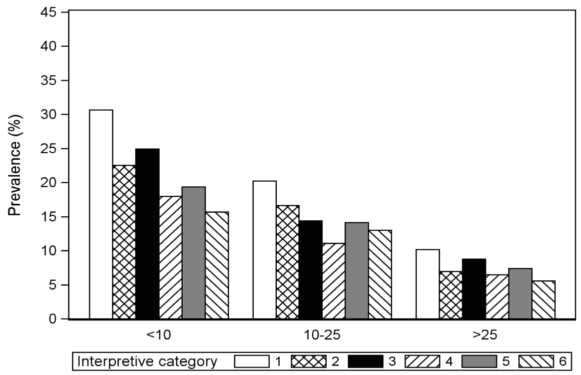

C. *M. catarrhalis,* Neutrophils per LPF

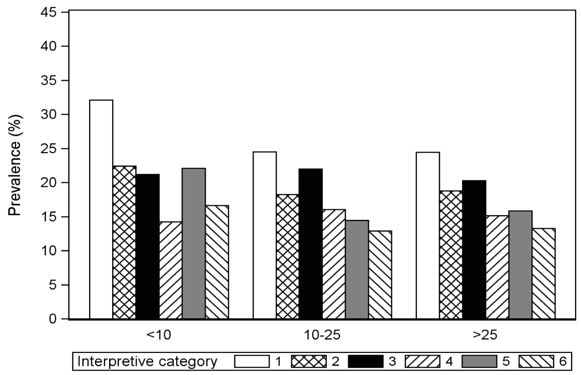

D. *S. aureus*, Epithelial cells per LPF

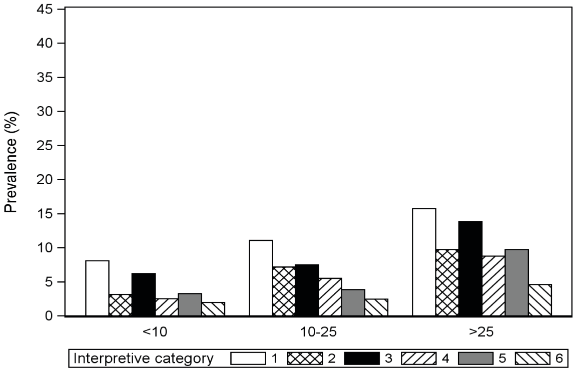

D. *S. aureus,* Neutrophils per LPF

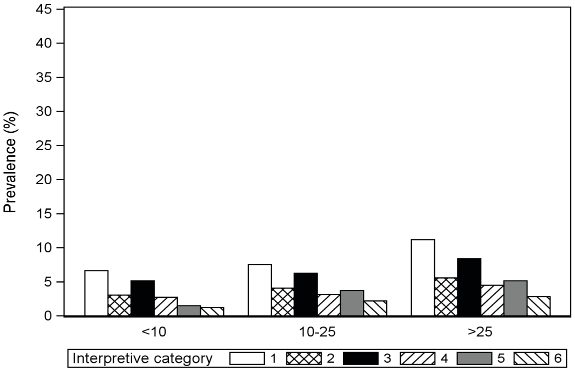

E. Other Gram-negative rods[b], Epithelial cells per LPF

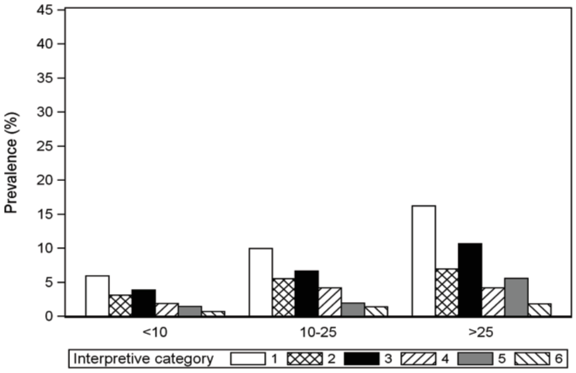

E. Other Gram-negative rods[b], Neutrophils per LPF

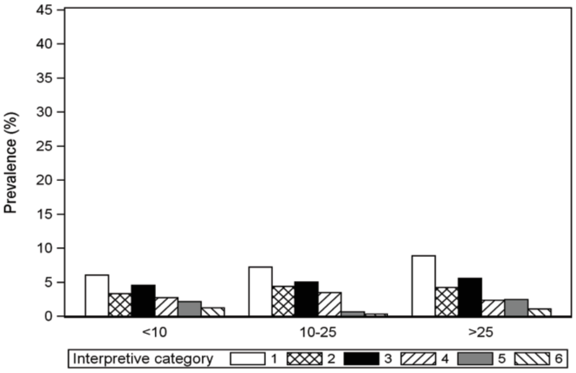

F. All organisms, Traditional IS quality measures

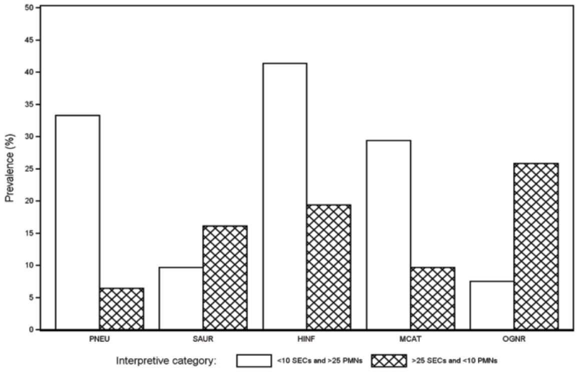

Abbreviations: HINF, *H. influenzae*;LPF, low power field; MCAT, *M. catarrhalis*; OGNR, Other non-fermentative gram-negative rods; PMNs, polymorphonuclear cells; PNEU, *S. pneumoniae*; SAUR, *S. aureus*; SECs, squamous epithelial cells; WHO, World Health Organization.

CXR+ defined as radiographic evidence of pneumonia (consolidation and/or other infiltrates) (N=1739).

Other non-fermentative gram-negative rods includes: *Acinetobacter* species and *Pseudomonas* species.

[a] Sputum interpretative criteria: 1 = organism present in any amount; 2 = present in any amount with compatible Gram stain morphotype; 3 = present as the predominant organism; 4 = present as the predominant organism with compatible Gram stain morphotype; 5 = present in quantities ≥=2+; 6 = present in quantities ≥=2+ with compatible Gram stain morphotype.

[b] Other non-fermentative gram-negative rods includes: *Acinetobacter* species and *Pseudomonas* species.

Supplementary Figure 1b. Prevalence of organisms by induced sputum culture interpretive criteria[a] and induced sputum quality variables in children aged 1-59 months with WHO-defined severe or very severe pneumonia, Cases with no prior antibiotics (N=842)

A. *H. influenzae*, Epithelial cells per LPF

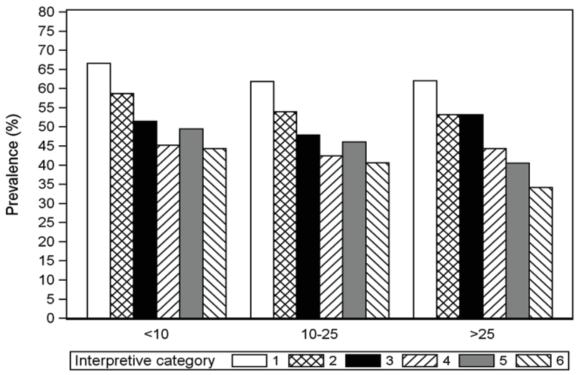

A. *H. influenzae*, Neutrophils per LPF

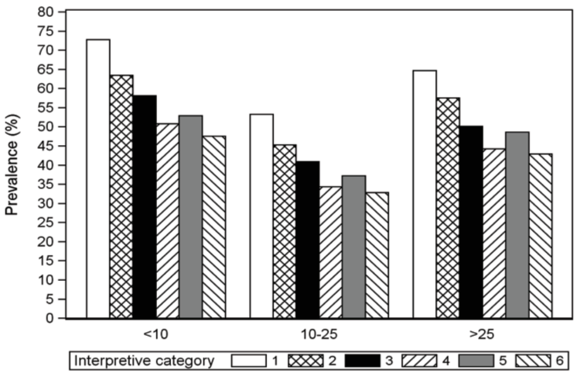

B. *S. pneumoniae*, Epithelial cells per LPF

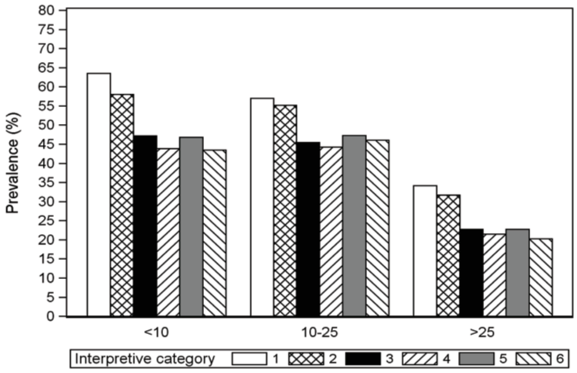

B. *S. pneumoniae,* Neutrophils per LPF

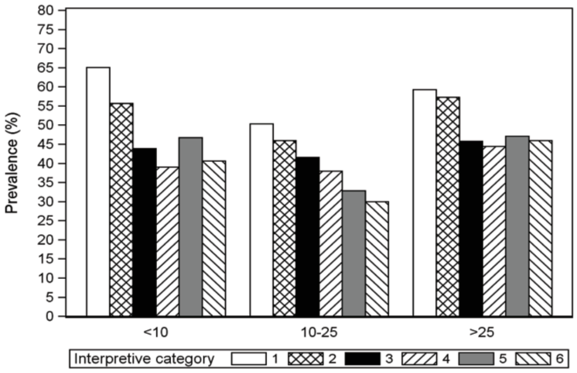

C. *M. catarrhalis*, Epithelial cells per LPF

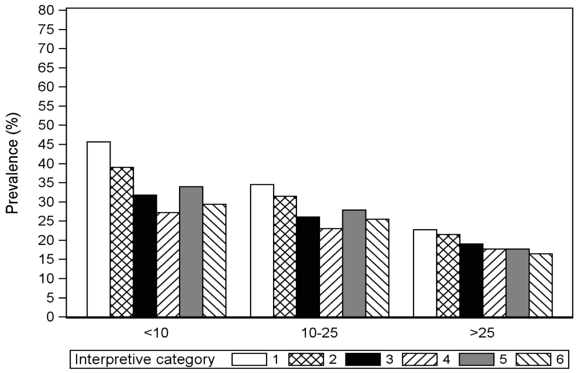

C. *M. catarrhalis,* Neutrophils per LPF

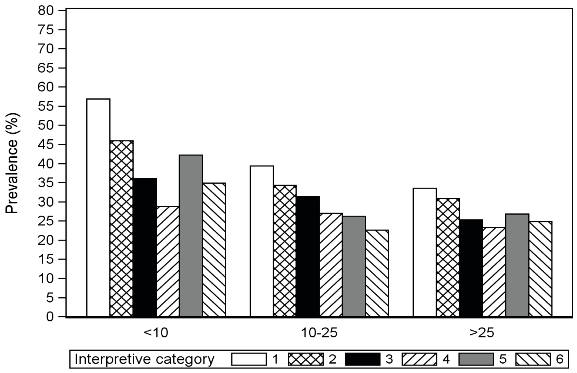

D. *S. aureus*, Epithelial cells per LPF

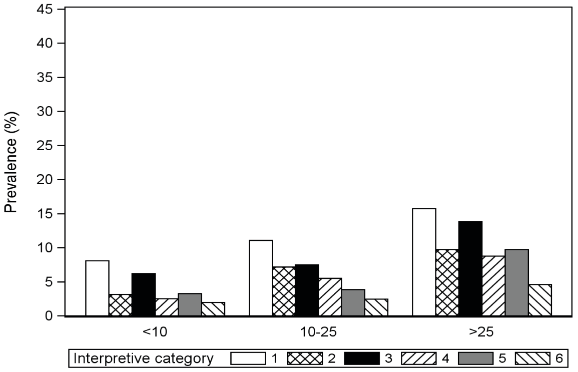

D. *S. aureus,* Neutrophils per LPF

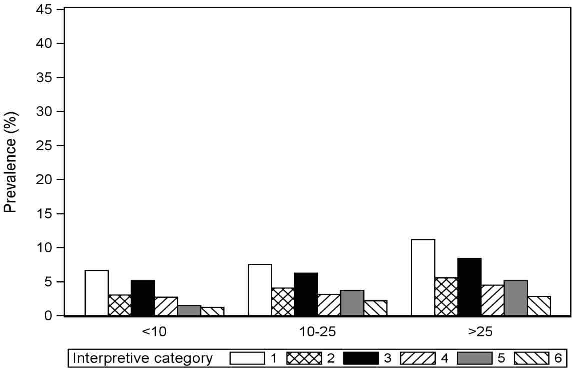

E. Other Gram-negative rods[b], Epithelial cells per LPF

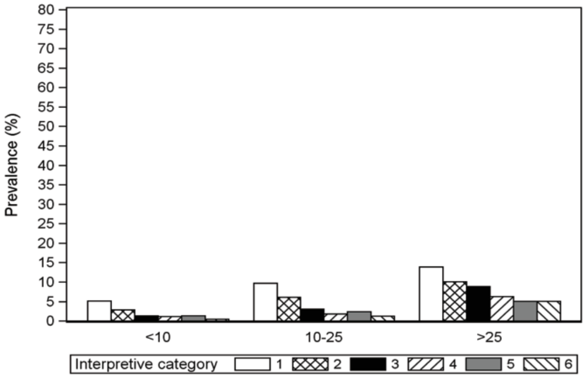

E. Other Gram-negative rods[b], Neutrophils per LPF

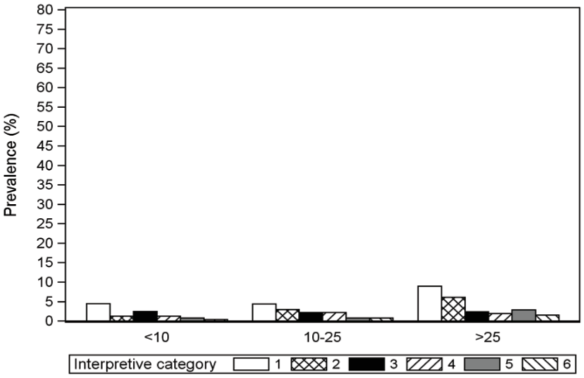

F. All organisms, Traditional IS quality measures

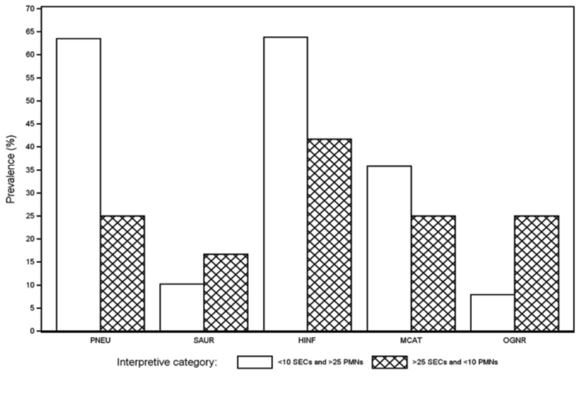

Abbreviations: HINF, *H. influenzae*; LPF, low power field; MCAT, *M. catarrhalis*; OGNR, Other non-fermentative Gram-negative rods; PMNs, polymorphonuclear cells; PNEU, *S. pneumoniae*; SAUR, *S. aureus*; SECs, squamous epithelial cells; WHO, World Health Organization.

Other non-fermentative gram-negative rods includes: *Acinetobacter* species and *Pseudomonas* species.

[a] Sputum interpretative criteria: 1 = organism present in any amount; 2 = present in any amount with compatible Gram stain morphotype; 3 = present as the predominant organism; 4 = present as the predominant organism with compatible Gram stain morphotype; 5 = present in quantities ≥=2+; 6 = present in quantities ≥=2+ with compatible Gram stain morphotype.

[b] Other non-fermentative gram-negative rods includes: *Acinetobacter* species and *Pseudomonas* species.

Supplementary Figure 1c. Prevalence of organisms by induced sputum culture interpretive criteria and induced sputum quality variables[a] in children aged 1-59 months with WHO-defined severe or very severe pneumonia, Cases with prior antibiotics (N=2833)

A. *H. influenzae*, Epithelial cells per LPF

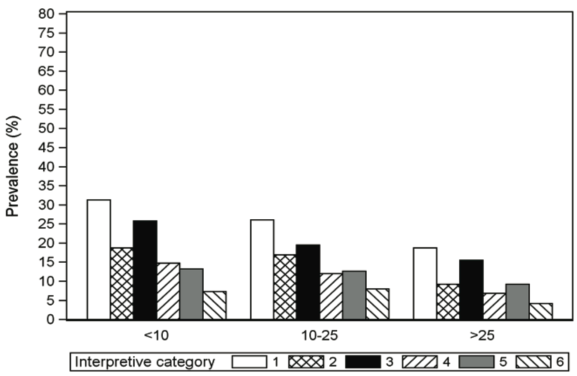

A. *H. influenzae*, Neutrophils per LPF

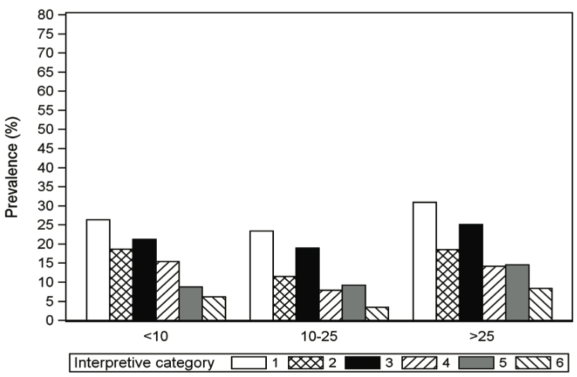

B. *S. pneumoniae*, Epithelial cells per LPF

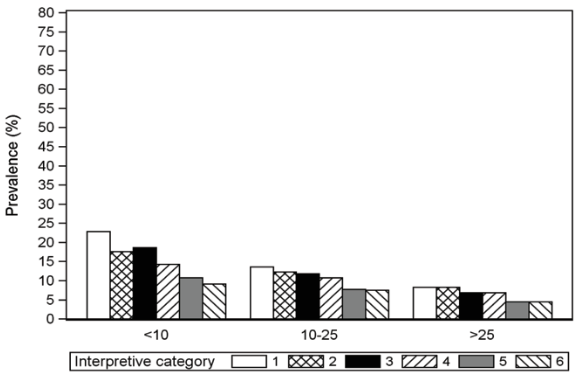

B. *S. pneumoniae,* Neutrophils per LPF

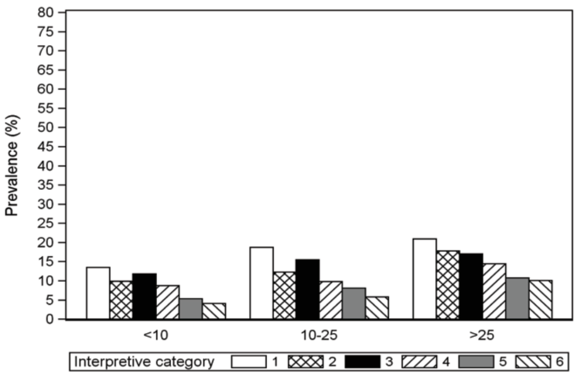

C. *M. catarrhalis*, Epithelial cells per LPF

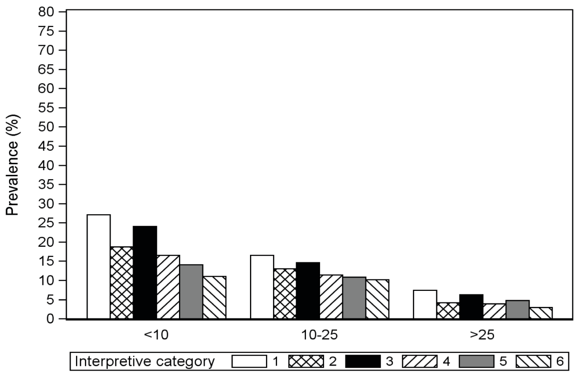

C. *M. catarrhalis,* Neutrophils per LPF

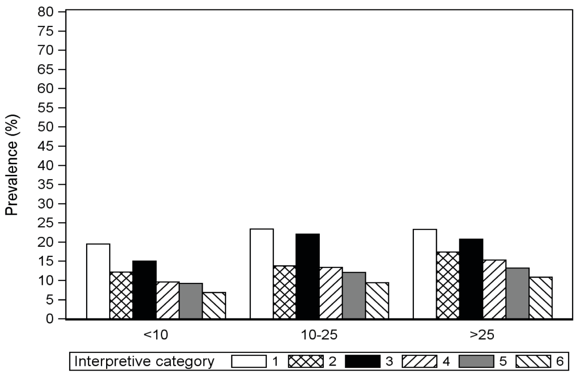

D. *S. aureus*, Epithelial cells per LPF

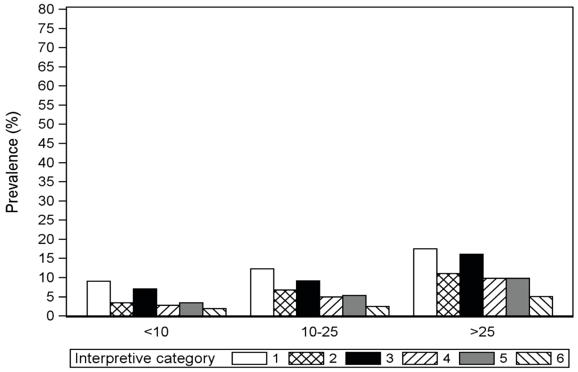

D. *S. aureus,* Neutrophils per LPF

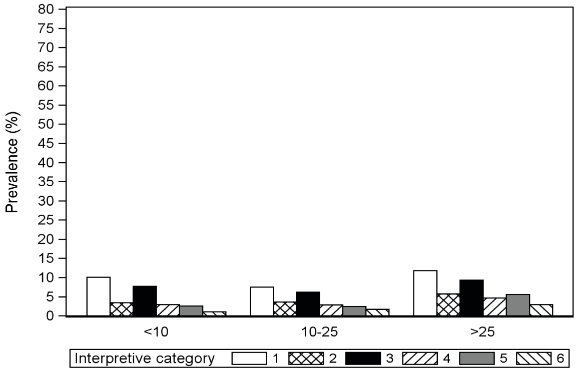

E. Other Gram-negative rods[b], Epithelial cells per LPF

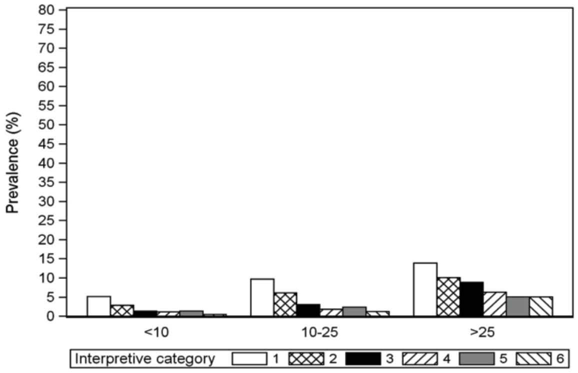

E. Other Gram-negative rods[b], Neutrophils per LPF

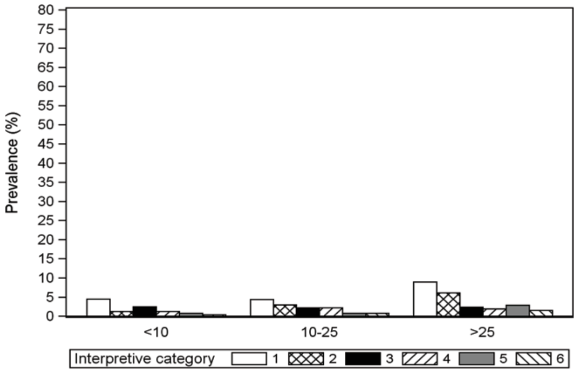

F. All organisms, Traditional IS quality measures

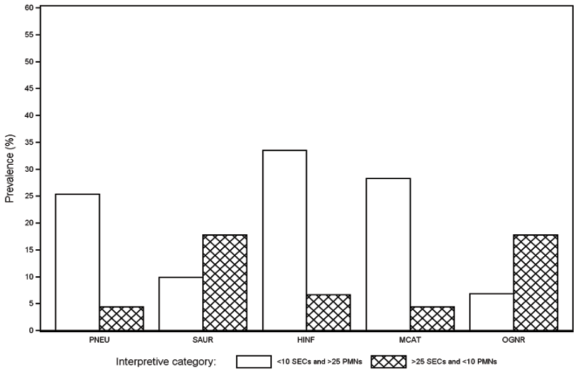

Abbreviations: HINF, *H. influenzae*; LPF, low power field; MCAT, *M. catarrhalis*; OGNR, Other non-fermentative Gram-negative rods; PMNs, polymorphonuclear cells; PNEU, *S. pneumoniae*; SAUR, *S. aureus*; SECs, squamous epithelial cells.
Other non-fermentative gram-negative rods includes: *Acinetobacter* species and *Pseudomonas* species.
[a] Sputum interpretative criteria: 1 = organism present in any amount; 2 = present in any amount with compatible Gram stain morphotype; 3 = present as the predominant organism; 4 = present as the predominant organism with compatible Gram stain morphotype; 5 = present in quantities ≥=2+; 6 = present in quantities ≥=2+ with compatible Gram stain morphotype.
[b] Other non-fermentative gram-negative rods includes: *Acinetobacter* species and *Pseudomonas* species.

Supplementary Figure 2a. Quantity of oropharyngeal flora in induced sputum in children aged 1-59 months with WHO-defined severe or very severe pneumonia by induced sputum quality variables, CXR+ cases (N=1739)

A. Epithelial cells per LPF

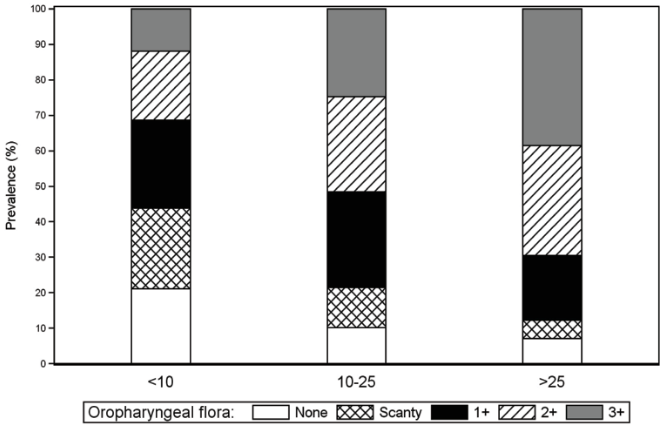

B. Neutrophils per LPF

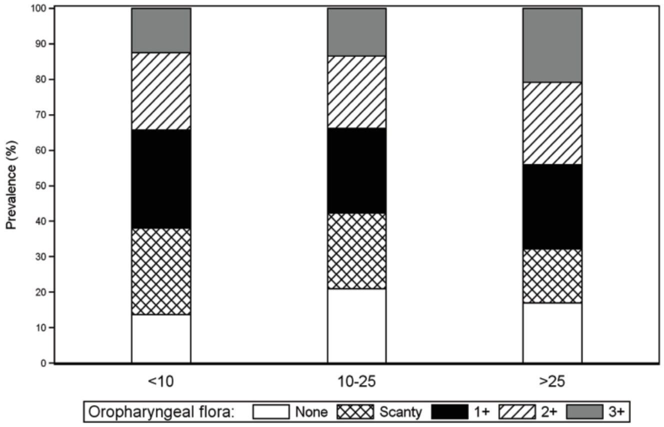

C. Traditional IS Quality Measures

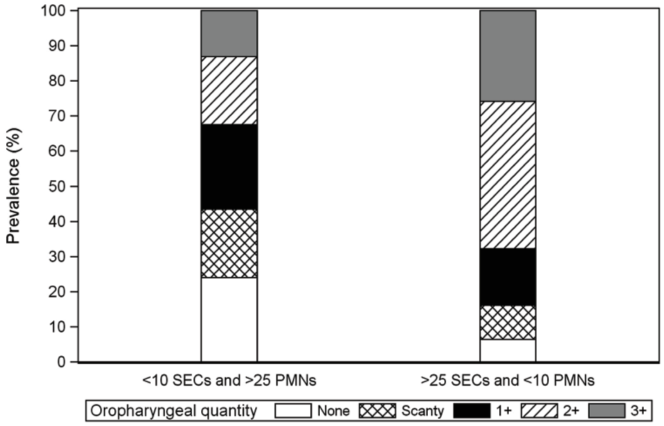

Abbreviations: LPF, low power field; PMNs, polymorphonuclear cells; SECs, squamous epithelial cells; WHO, World Health Organization.
CXR+ defined as radiographic evidence of pneumonia (consolidation and/or other infiltrates).

Supplementary Figure 2b. Quantity of oropharyngeal flora in induced sputum in children aged 1-59 months with WHO-defined severe or very severe pneumonia by induced sputum quality variables, Cases with no prior antibiotics (N=842)

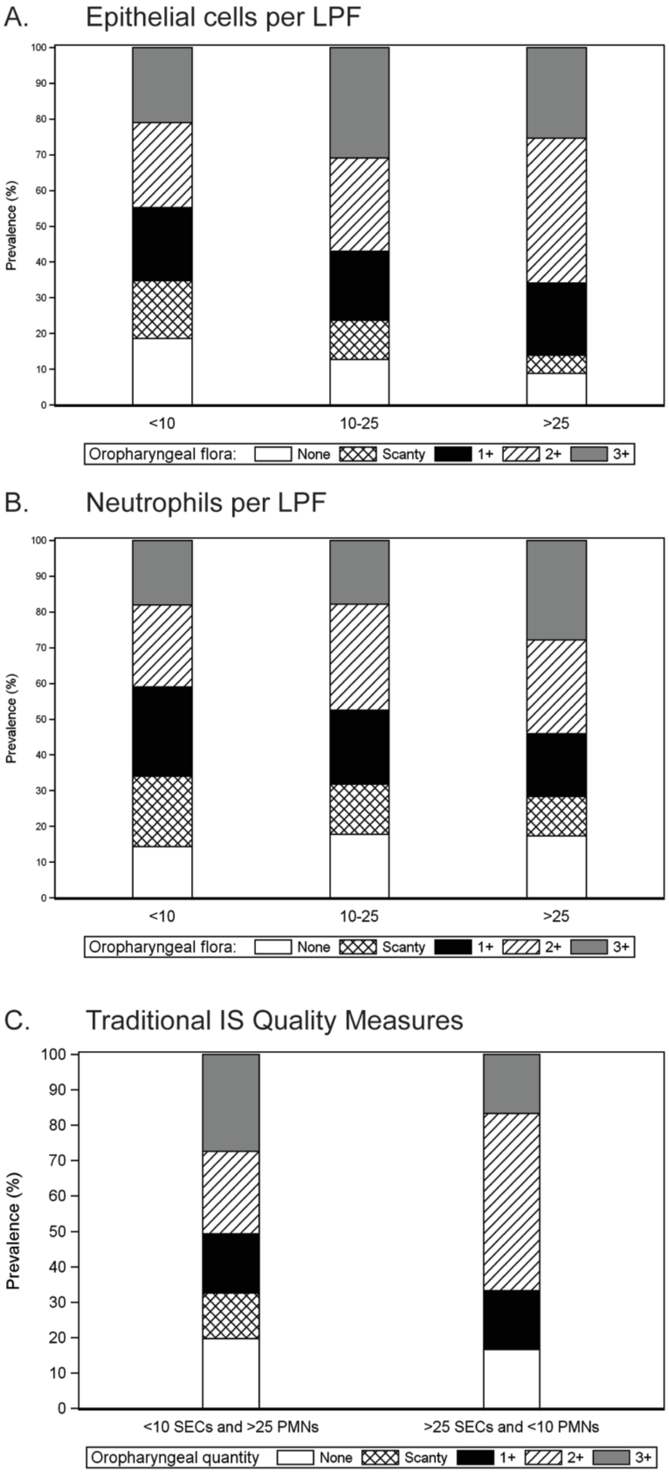

Abbreviations: LPF, low power field; PMNs, polymorphonuclear cells; SECs, squamous epithelial cells; WHO, World Health Organization.

Supplementary Figure 2c. Quantity of oropharyngeal flora in induced sputum in children aged 1-59 months with WHO-defined severe or very severe pneumonia by induced sputum quality variables, Cases with prior antibiotics (N=2833)

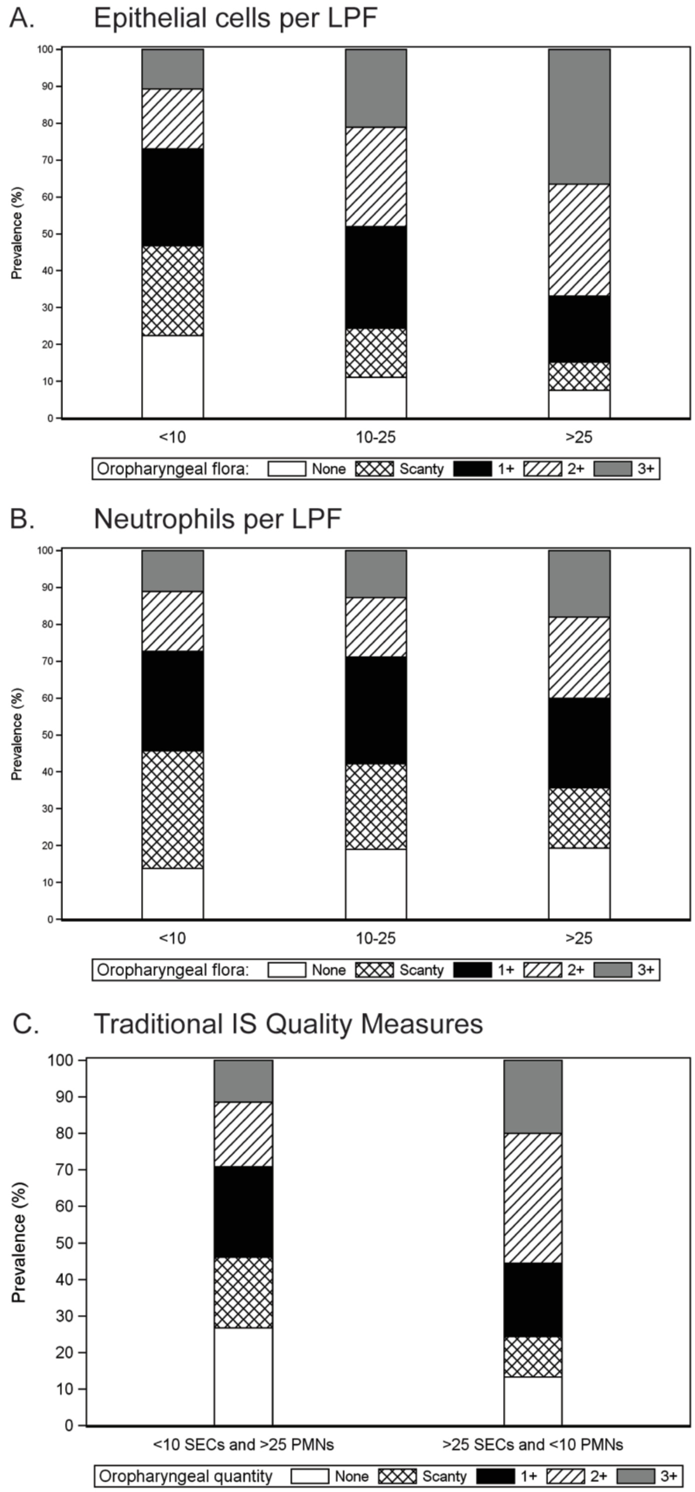

Abbreviations: LPF, low power field; PMNs, polymorphonuclear cells; SECs, squamous epithelial cells; WHO, World Health Organization.

Acknowledgements:
PERCH Expert Group. William C. Blackwelder, Harry Campbell, John A. Crump, Adegoke Falade, Menno D. de Jong, Claudio Lanata, Kim Mulholland, Shamim Qazi, Cynthia G. Whitney.

Pneumonia Methods Working Group. Robert E Black, Zulfiqar A Bhutta, Harry Campbell, Thomas Cherian, Derrick W Crook, Menno D de Jong, Scott F Dowell, Stephen M Graham, Keith P Klugman, Claudio F Lanata, Shabir A Madhi, Paul Martin, James P Nataro, Franco M Piazza, Shamim A Qazi, and Heather J Zar.

PERCH Chest Radiograph Reading Panel
Readers: Dr. Kamrun Nahar, Dr. Fariha Bushra Matin, Dr. Claire Oluwalana, Dr. Bernard Ebruke, Dr. Joyce Sande, Dr. Micah Silaba, Dr. Mahamadou Diallo, Dr. Breanna Barger-Kamate, Dr. Nasreen Mahomed, Dr. David Moore, Dr. Anchalee Kruatrachue, Dr. Piyarat Suntarattiwong, Dr. Musaku Mwenechanya, Dr. Rasa Izadnegahdar, **Arbitrators:** Dr. Vera Manduku, Dr. John DeCampo, Dr. Marg DeCampo, Dr. Fergus Gleeson.

PERCH Contributors:
Bangladesh: Kamrun Nahar, Arif Uddin Sikdir, Sharifa Yeasmin, Dilruba Ahmed, Muhammad Ziaur Rahman, Muhammad Yunus, Muhammad Al Fazl Khan, Muhammad Jubayer Chisti, Abu Sadat Muhammad Sayeem, Shahriar Bin Elahi, Mustafizur Rahman; **The Gambia:** Michel Dione, Emmanuel Olutunde, Peter Githua, Ogochukwu Ofordile, Rasheed Salaudeen, David Parker; **Kenya:** Shebe Mohamed, Siti Ndaa, Micah Silaba, Neema Muturi, Angela Karani, Sammy Nyongesa, Anne Bett, Daisy Mugo, Salim Mwarumba, Robert Musyimi, Andrew Brent, James Nokes, David Mulewa, Joyce Sande, John Odhiambo, Joshua Wambua, Nuru Kibirige, Caroline Mulunda, Hellen Mjalla, Norbert Katira, Karen Dama, Loice Masha, Christine Mutunga, Mwanajuma Ngama, Stephen Mangi, Riziki Anthony, Mwarua Yubu, Elijah Wakili, Benson Katana, Shoboi Mgunya, Emmanuel Mumba, Benedict Mver, George Kuria, Felix Githinji, Norbert Kihuha, Boniface Jibendi, Tahreni Bwanaali, Agustus Kea; **Mali:** Nana Kourouma, Aliou Toure, Mahamadou Diallo, Breana Barger-Kamate, Mariam Samake, Seydou Sissoko, Abdoul Aziz Maiga, Mariam Samake, Toumani Sidibe, Mariam Sylla, Aziz Diakite, Bassirou Diarra; **South Africa:** Azwidihwi Takalani, Andrea Hugo, Susan Nzenze, Ndulela Titi, Mmabatho Selela, Malebo Motiane, Minah Nkuna, Nonhlanhla Tsholetsane, Sibonsile Moya, Debra Katisi, Tondani Netshishivhe, Lerato Mapetla, Gudani Singo, Simphiwe Gasa, Cece Mgenge, Nozipho Mthunzi, Nombulelo Monedi, Tanja Adams, Shafeeka Mangera, Jeannette Wadula, Peter Tsaagane, Jenifer L.

Vaughan, Sakina Loonat, Martin Hale, Sugeshnee Pather, Mariëtte Middel, Siobhan Trenor, Palesa Morailane, Ntombi Maya, Rene Sterley, Charné Combrinck, Given Malete, Lerato Qoza, Grizelda Liebenberg, Hendrik van Jaarsveld, Zunaid Kraft, Lisa-Marie Mollentze, Lourens Combrinck, Tsholofelo Mosome; **Thailand:** Sununta Henchaichon, Dr. Tussanee Amornintapichet, Dr. Somchai Chuananont, Toni Whistler, Juraiporn Ratanodom, Patranuch Sapchookul, Ornuma Sangwichian, Sirirat Makprasert, Manoon Hirunsalee, Possawat Jorakate, Anek Kaewpan, Duangkamol Siludjai, Apiwat Lapamnouysup, Dr. Wantana Paveenkittiporn, Waraporn Ubonphen, Dr. Peera Areerat, Ms.Yupapan Wannachaiwong, Ms Tewa Faipet, Ms Punnat Natnarakorn, Ms Ahchanan Sacharone, Mr.Winai Makmool, Ms. Kanlaya Sornwong, Ms. Promporn Sansuriwong, Ms. Ratchanida Potiya, Ms. Wasana Hongsawong, Ms.Wipa Matchaikhen, Ms. Thatsanawan Chaiyabil, Ms.Piyapai Wannarach, Ms Chamaiporn Wadeesirisak, Mr. Yuttapong Norapet, Mattana Bangkung, Mr. Barameht Piralam, Sathapana Naorat, Anchalee Jatapai, Prasong Srisaengchai, Dr. Leonard Peruski, Ms.Dawan Phaensoongnoen, Ms.Tussaaorn Klangprapan, Ms.Narawadee Dumrongdee, Ms.Atchara Srithongkham, Mr. Piyawut Noinont, Ms. Pornthip Kamlee, Ms.Siyapa Mongkornsuk; **Zambia:** Justin Mulindwa, Musaku Mwenechanya, John Mwaba, Magdalene Mwale, Julie Duncan, Kazungu Siazele, Muntanga Mapeni, Emily Hammond; **Canterbury Health Laboratory, Christchurch, New Zealand:** Rose Watt, Shalika Jayawardena; **The Emmes Corporation, Rockville, Maryland:** Mark Wolff, Megan Sanza, Omid Neyzari.
